# Electrochemical Detection of Aβ42 and Aβ40 at Attomolar Scale via Optimised Antibody Loading on Pyr-NHS-Functionalised 3D Graphene Foam Electrodes

**DOI:** 10.3390/bios15120806

**Published:** 2025-12-10

**Authors:** Muhsin Dogan, Sophia Nazir, David Jenkins, Yinghui Wei, Genhua Pan

**Affiliations:** 1 Nanomaterials and Devices Laboratory (NMD), School of Engineering, Computing and Mathematics, University of Plymouth, Devon PL4 8AA, UK; 2 Biomedical Engineering, Engineering and Architecture Faculty, Izmir Bakircay University, Izmir 35665, Turkey; 3 School of Engineering, Computing and Mathematics, University of Plymouth, Devon PL4 8AA, UK

**Keywords:** Alzheimer’s disease (AD), electrochemical biosensor, amyloid beta, 3D graphene foam, Pyr-NHS

## Abstract

Alzheimer’s Disease (AD) is one of the most commonly seen neurodegenerative disorders, where early detection of its biomarkers is crucial for effective management. Conventional diagnostic methods are often expensive, time-consuming, and highly complex, which highlights an urgent need for point-of-care biosensing technology. In this work, we developed assays on three-dimensional (3D) graphene foam electrodes by functionalising them with a 1-Pyrenebutyric acid N-hydroxysuccinimide ester (Pyr-NHS) to enable effective antibody immobilisation for the detection of amyloid beta peptides (Aβ42 and Aβ40), key biomarkers for AD. Pyr-NHS linkers were used for stable functionalisation, followed by binding with Aβ42 and Aβ40 antibodies, and then bovine serum albumin (BSA) was employed as a blocking agent to minimise non-specific bindings on the electrode surface. Differential Pulse Voltammetry (DPV) measurements showed satisfactory stability over 12 days (RDS upper limit was <10%) and highly sensitive and specific detection of Aβ42 and Aβ40, with insignificant interference of tau217 protein. The biosensor exhibited a low limit of detection (LOD) with 252 aM for Aβ42 and 395 aM for Aβ40, covering 0.125 fM–1 nM and 0.125 fM–100 pM linear ranges, respectively. Further validation was conducted on spiked-diluted human plasma. This excellent analytical performance was attributed to the stable Pyr-NHS functionalisation, the 3D graphene foam enabling superior conductivity and a larger surface area on the working electrode, and the optimisation of antibody concentration for immobilisation. These promising results suggest that 3D graphene foam-based biosensors have considerable potential for early detection of AD biomarkers and developing cost-effective, portable, and reliable point-of-care devices.

## 1. Introduction

Dementia is a disorder of the brain that show its symptoms with ageing. Approximately 55 million people are living with it, and the number of cases is gradually increasing, estimated to reach 140 million by 2050. Its socioeconomic effect is expected to rise considerably as well, which was USD 1.3 trillion in 2019 and is estimated to increase by USD 1.5 trillion by 2030. Alzheimer’s Disease (AD), which impacts cognitive abilities such as thinking, remembering, and decision-making, is the most common cause of dementia, accounting for roughly 65 percent of cases [1]. Despite the high number of global cases, there is no approved cure for AD [2]. The main reason for this situation is that AD diagnosis is mostly carried out when AD reaches a severe level [3].

Amyloid plaques formed by the accumulation of amyloid beta peptides between neurons and neurofibrillary tangles, which are aggregates of the tau protein in neurons, are considered the main incidents that trigger AD pathogenesis [4]. The pathogenesis of AD can occur years before its initial symptoms appear [5,6]. To develop an effective treatment for AD, therefore, it is crucial to detect the pathological changes caused by AD before the first AD symptoms occur [2].

It is difficult to distinguish the pathological changes caused by AD because the condition develops slowly. To overcome this difficulty, involving biomarkers, medical signs caused by pathological changes, drugs, or diseases, in AD diagnostic applications is important. In other words, biomarkers play a vital role in the diagnosis of AD and in devising effective management methods for the disease [7]. Amyloid beta peptides (such as Aβ42 and Aβ40), Tau protein (such as P-tau 181 and P-tau 217), and Apolipoprotein E4 (APOE4) have been commonly used for AD biomarker detection [8].

Conventionally, cerebrospinal fluid (CSF) is the centre of AD diagnostic methods. Positron emission tomography (PET) and some invasive CSF collection methods are used to detect AD. However, these methods can be considered invasive, time-consuming, and expensive. Additionally, these methods are not easily accessible [8]. Therefore, blood sampling can be a better alternative because it is less complicated, expensive, and invasive for AD biomarker detection compared to CSF collection and PET imaging [3].

Electrochemical biosensors can provide quicker and more cost-effective methods for biomarker detection. They are also user-friendly, easily accessible, and less invasive. These features make electrochemical biosensors effective tools for AD biomarker detection [9,10]. Sethi et al. [11] developed assays on reduced graphene oxide (rGO) screen-printed electrodes to detect Aβ42 and Aβ40. They used NH_2_ as a linker to improve sensitivity and protein G to enhance the binding between the linker and the antibodies. The limit of detection (LOD) of the biosensor was reported as 8.65 fM for Aβ1-42 and 9.51 fM for Aβ1-40. Devi et al. [12] developed a chitosan nanocomposite-modified glassy carbon electrode to detect Aβ1-42 peptides. To enhance the sensing performance of the electrodes, gold nanoparticle/nickel ferrite-modified graphene oxide was produced. The LOD of the study was highlighted as 3 pg/mL. However, recent studies have not shown good sensitivity and LOD levels, since AD biomarkers can be found in blood at very low concentrations (tens of pg/mL [13]). For this reason, developing sensitive, cost-effective, and reliable biosensors that can detect low-level concentrations of AD biomarkers is significant for managing early detection of AD.

Graphene, comprising a single layer of carbon atoms arranged in a 2D (two-dimensional) honeycomb pattern, is a groundbreaking material. It possesses extraordinary characteristics including excellent electrical conductivity, flexibility, remarkable strength, and convenience of functionalising its surface. Gii-Sens graphene electrodes are in the form of 3D (three-dimensional) foam. This feature provides them a 25% larger electrochemically active area compared to other 2D (two-dimensional) electrochemical graphene sensors. Another advantage of Gii-Sens electrodes is that they are highly pure in carbon, roughly 98%, which is significant for electrochemical biosensor applications [14]. Some studies in the literature indicated that Gii-Sens electrodes offer effective sensing performances in biosensor applications [15,16,17].

In this study, we report the assay development on a 3D graphene-based electrochemical biosensor and its evaluation for the detection of AD blood biomarkers. The sensor was functionalised using antibodies specific to Aβ42 and Aβ40 peptides, with bovine serum albumin (BSA) employed as a blocking agent to prevent non-specific binding. The surface morphology and successful functionalisation were characterised using Scanning Electron Microscopy (SEM) and Fourier Transform Infrared Spectroscopy (FTIR) (Bruker Corporation, Billerica, MA, USA), while the electrochemical performance of the biosensor was evaluated through Differential Pulse Voltammetry (DPV). The sensor exhibited a low limit of detection, high sensitivity, and strong selectivity and specificity toward its target biomarkers, demonstrating its potential as a reliable tool for early AD diagnosis.

## 2. Materials and Methods

### 2.1. Reagents and Apparatus

Aβ42 antibody (H31L21) was purchased from Thermo Fisher Scientific (Paisley, UK), whereas Aβ40 antibody was obtained from Bio-legend (London, UK). Aβ42 human peptide, phosphate-buffered saline (PBS) tablets, bovine serum albumin (BSA), potassium ferricyanide (K_3_Fe(CN)_6_), and potassium chloride (KCl) were bought from Sigma Aldrich (Gillingham, UK), and Aβ40 human peptide was purchased from Tocris Bioscience (Abingdon, UK). P-tau217 peptides were purchased from Abcam (Cambridge, UK). Real human plasma with K_2_EDTA as anticoagulants was purchased from Cambridge Bioscience (Cambridge, UK). 1-Pyrenebutyric acid N-hydroxysuccinimide ester (Pyr-NHS) was bought from Lumiprobe (Hannover, Germany), and N,N-Dimethylformamide (DMF) was purchased from Fisher Scientific (Loughborough, UK).

Gii-Sens Integrated Graphene electrodes were purchased from iGii (Stirling, UK) and used as sensors. These electrodes have 3D graphene foam as the working electrode (4 mm diameter), a counter electrode, and a reference electrode made of Ag/AgCl (Figure S1). To perform electrochemical measurements, a Dropsens µstat ECL potentiostat purchased from Metrohm Dropsens (Runcorn, UK) was used. DropView 8400 2.0 software was used to set measurement methods and parameters and to visualise the measurement results. For statistical analysis and plotting of the results, SigmaPlot 16.0 software was used. To perform surface characterisation experiments, Scanning Electron Microscope (LV-SEM, Jeol, 6610, Tokyo, Japan) and Fourier Transform Infrared Spectroscopy (Alpha-P) techniques were carried out. To obtain the desired temperature for the experiments, a MyTemp mini-incubator (Benchmark Scientific, Sayreville, NJ, USA) was used.

### 2.2. Electrochemical Measurements

All electrochemical measurements were conducted at room temperature using the incubator. To prepare the electrolyte, 10 mM potassium ferricyanide was used with 1 M KCI solution as the supporting chemical. Cyclic Voltammetry (CV) and Differential Pulse Voltammetry (DPV) were used to analyse the electrochemical performance and analytical performance of the biosensor. For each measurement, 100 µL of electrolyte was dropped on the electrode surface. The scan rate for CV was set to 50 mV s^−1^ from 0.2 V to −0.4 V, and the Estep was 1 mV. DPV was conducted at a 50 mV s^−1^ scan rate from −0.4 V to 0.2 V, with a pulse amplitude of 50 mV, a pulse period of 40 ms, and a step potential of 10 mV.

### 2.3. Preparation of the Electrodes for Experiments

First, the bare Gii-Sens 3D electrodes were functionalised with Pyr-NHS. To do this, 10 mM Pyr-NHS was obtained by mixing it with 90% DMF (90:10 DMF/water). Then, 10 μL of Pyr-NHS solution was dropped on each electrode, and the electrodes were kept at 20 °C in the incubator overnight.

**Figure 1:**
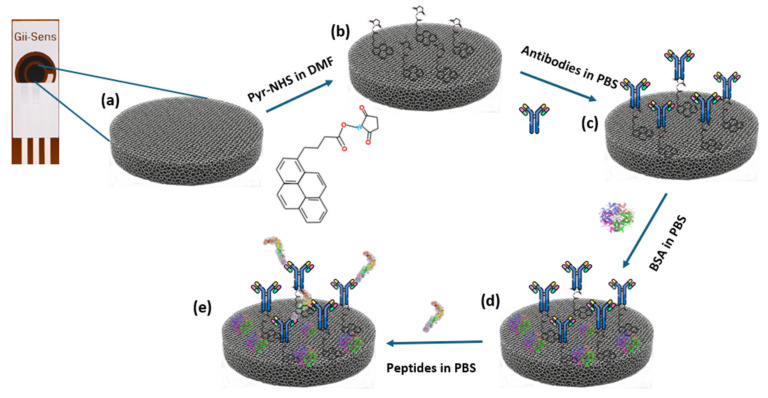
Diagram of the fabrication of the biosensor. (**a**) Bare 3D graphene foam working electrode. (**b**) Illustration of the electrode surface after Pyr-NHS treatment. (**c**) Antibody immobilisation. (**d**) Involving BSA in the process as a blocking agent. (**e**) Representative illustration of the biomarker detected by the antibodies.

After Pyr-NHS functionalisation, 10 μL of the solution with the antibodies, prepared in PBS with the desired concentration (30 μg/mL), was dropped on the working electrode, and they stayed in the incubator for 120 min at room temperature. Then, the electrodes were washed with PBS. BSA was used after these steps as a blocking agent. The BSA solution was prepared in PBS at a 0.5% ratio. After that, 10 μL of the BSA solution was dropped on the electrodes at room temperature, and they were left for 15 min. Thereafter, PBS was used to wash the electrodes to make them ready for the biorecognition process. The concept of the fabrication steps is depicted in Figure 1.

### 2.4. Biosensor–Antigen Interaction

PBS was used to prepare a wide range of concentration levels of the antigens (Aβ42 and Aβ40) by separately mixing each antigen in PBS using a vortex for 30 s. Then, 10 μL of each antigen solution was dropped on the electrodes, and they were incubated for 60 min at room temperature. After that, the electrodes were washed with PBS to remove unbound antigens on the electrode surface. Finally, the electrodes became ready for the electrochemical characterisation measurements.

### 2.5. Preparation of Spike-Diluted Plasma Samples

The purchased human plasma was diluted at the ratio of 1:100 using PBS. After that, the desired target antigen amount was added and mixed via a vortex mixer for 30 s. By performing this, the desired concentration levels were obtained. Subsequently, 10 μL of each antigen solution was dropped on the electrodes, and they were incubated at room temperature for 60 min. Finally, the sensors were washed with PBS and then they were ready for the DPV measurements.

## 3. Results

### 3.1. Characterisation of Stepwise Functionalisation of the 3D Graphene Foam Electrode Surface

#### 3.1.1. FTIR Analysis

**Figure 2:**
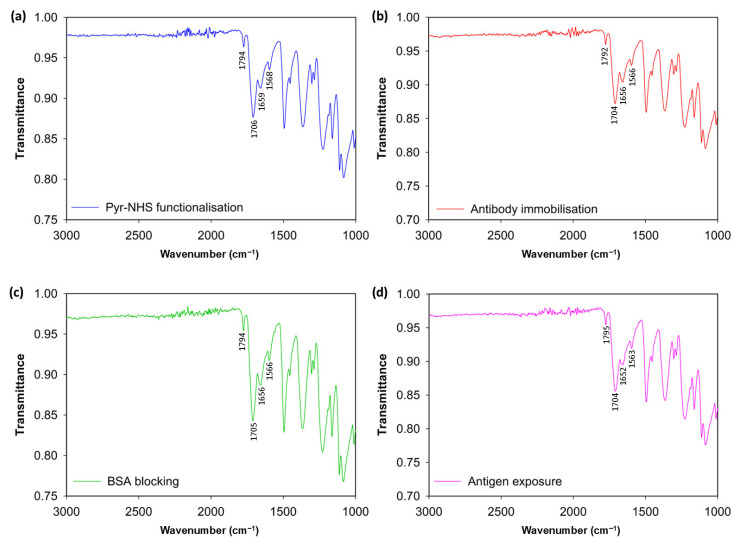
FTIR spectrum results. Modification of the bare electrode surface with Pyr-NHS linker (**a**), antibody immobilisation after the linker (**b**), BSA blocking (**c**), and antigen introduction to the electrodes (**d**).

To analyse the stepwise functionalisation of the 3D graphene electrode surface, FTIR was conducted (Figure 2). The initial modification caused by Pyr-NHS demonstrated its characteristic absorption band at ~1700 and ~1800 cm^−1^ (Figure 2a), which is related to the symmetric and asymmetric stretching of the NHS ester carbonyl groups (C=O–O–N) [17]. After the covalent immobilisation of the antibodies (Figure 2b), the changes were modest: the carbonyl region stayed pronounced, and slight increases in ≈1650 cm^−1^ (amide I, C=O) and ≈1560 cm^−1^ (amide II, N–H/C–N) indicated the presence of protein [18,19]. Subsequently, blocking with BSA reinforced this protein signature, indicating effective passivation of remaining sites (Figure 2c). Finally, introducing antigens to the electrode surface resulted in additional broadening and slight enhancement of the amide bands (Figure 2d), indicative of increased protein mass; however, these characteristics remain partially obscured by linker vibrations and diminished by substrate scattering. As a result, the FTIR data support the expected stepwise chemistry qualitatively. However, the strong background from the 3D graphene foam and baseline distortion due to scattering hinder FTIR from demonstrating definitive, independent evidence of covalent antibody/antigen attachment. Therefore, these findings are most effectively presented as supplementary evidence alongside more surface-specific (XPS/ToF-SIMS) or functional (CV, DPV) validations. The functional validation of the successful fabrication of the biosensor is provided in the Section 3.2.

#### 3.1.2. SEM Imaging

**Figure 3:**
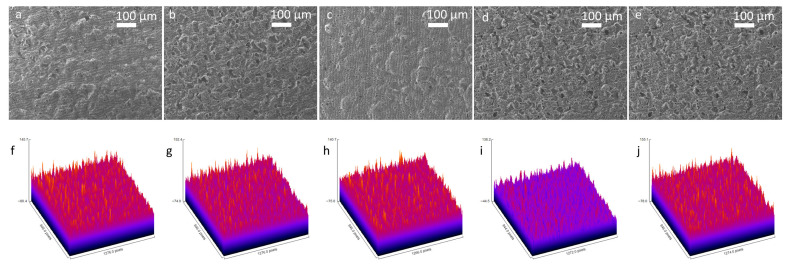
SEM images of the stepwise fabrication of the biosensor with 100× magnification and 100 μm scale and roughness plots. SEM image of the bare 3D graphene foam (**a**) and its roughness plot (**f**). SEM image after Pyr-NHS functionalisation (**b**) and its roughness plot (**g**). Following SEM image with antibody immobilisation (**c**) and its roughness plot (**h**). BSA blocking SEM image (**d**) and its roughness plot (**i**). Finally, the SEM image taken after antigen capture (**e**) and its roughness plot (**j**).

Scanning Electron Microscopy (SEM) was conducted with a 100 μm scale to examine morphological changes on the 3D graphene foam electrode surface throughout the biosensor fabrication and biorecognition, which included the sequential functionalisation of the bare electrode with Pyr-NHS, antibody immobilisation, and BSA blocking, and finally antibody capturing (Figure 3a–e). To analyse and compare roughness changes after each step, ImageJ 1.54p software was used to obtain roughness plots (Figure 3f–j) and Root Mean Square Roughness (R_q_). The sampling length was determined as 100 μm. The SEM image of the bare 3D graphene foam electrode (Figure 3a) exhibited a porous and interconnected structure, which provides a high surface area, and the roughness plot of the bare electrode (Figure 3f) supported this fact with a high ~226.1 AU Z-axis amplitude (peak-to-valley distance) and an R_q_ value of 20.07 AU. After Pyr-NHS functionalisation, the SEM image (Figure 3b) and the roughness plot (Figure 3g) showed that the functionalisation added a very thin layer on the surface, but the surface structure was not significantly affected, and the Z-axis amplitude was ~226.4 AU and the R_q_ was 22.4 AU. This change can be attributed to the thin non-covalent π–π stacking and the NHS ester. With antibody immobilisation (Figure 3c,h), the electrode exhibited less porosity and a smoother surface with ~215.7 AU of Z-axis amplitude and ~18.32 AU of R_q_, caused by the relatively large structure of the antibodies (~10 nm) masking the electrode surface by binding the Pyr-NHS. BSA blocking caused maximal planarisation on the surface, achieving the lowest Z-range (~182.7 AU) and R_q_ (~15 AU), confirming optimal passivation of non-specific binding sites (Figure 3d,i). The final step of antigen capture (Figure 3e) caused a dramatic increase in roughness, with the highest Z-axis amplitude (~233.1 AU) and R_q_ (~23.87 AU), confirming the bulky antibody–antigen formation on the surface. Thus, this correlational analysis of the SEM images supported the validity of the structural integrity and the stepwise functionalisation of the biosensor.

### 3.2. Electrochemical Analysis

**Figure 4:**
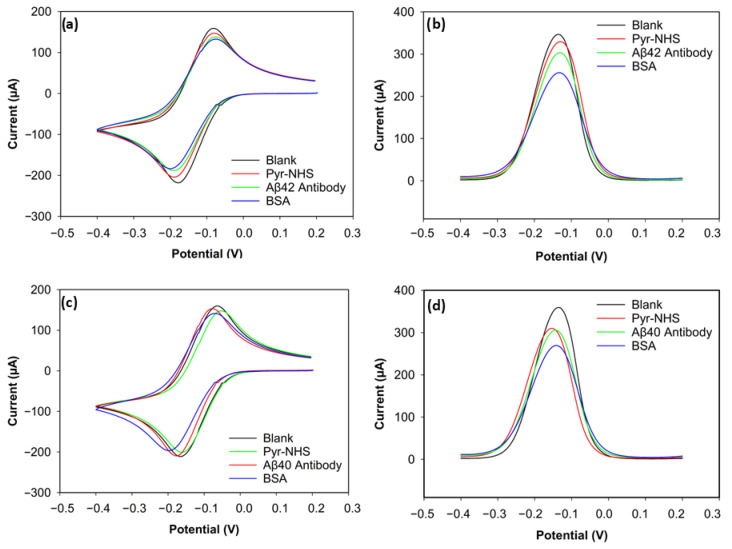
CV and DPV results of Aβ42 and Aβ40 electrodes. (**a**) CV voltammogram of Aβ42. (**b**) DPV voltammogram of Aβ42. (**c**) CV voltammogram of Aβ40. (**d**) DPV voltammogram of Aβ40.

The stepwise electrochemical analysis of biosensor fabrication was carried out with CV and DPV (Figure 4). For the CV voltammograms (Figure 4a,c), slight changes in the redox peaks were observed for electrodes functionalised for biorecognition of Aβ42 and Aβ40. The subtle changes in peak currents indicated charge-transfer resistance changes and hindered electrode transfer caused by non-conductive layers built on the electrode surface. The reason CV showed low sensitivity was that its capacitive background does not allow it to achieve a satisfactory signal-to-noise ratio [20]. On the other hand, the DPV results of the biofunctionalisation exhibited relatively high sensitivity in showing changes in electroactivity during the stepwise functionalisation of biosensors for both Aβ42 and Aβ40 (Figure 4b,d), which can be attributed to a better signal-to-background ratio provided by DVP [21].

#### 3.2.1. Electrochemical Characterisation of Aβ42 Biosensor

The positive peak current (the anodic peak current) of the blank electrode for Aβ42 reached 158 µA (Figure 4a). After Pyr-NHS treatment, the anodic (positive) peak decreased to 147 µA. A similar change can be seen in the cathodic peaks. The cathodic (negative) peak of the blank electrode was −218 µA, and the cathodic peak after Pyr-NHS was −204 µA. This decrease in electron transfer between the electrode and the electrolyte can be attributed to the organic layer caused by the pyrene [21]. A similar trend was observed after antibody immobilisation. The anodic peak dropped to 137 µA, and the cathodic peak current became −187 µA, which can be caused by the antibodies hindering electron transfer between the electrode and the electrolyte because of their large biomolecular structure [22]. BSA, a blocking agent that covers unoccupied areas on the surface after the linker and antibody immobilisation to prevent non-specific bindings, also caused a slight decrease in peak currents by 5 µA in the anodic and 4 µA in the cathodic peaks, which became 132 µA and −183 µA, respectively, due to its non-conductive nature [23].

The DPV result of the stepwise functionalisation for Aβ42 showed a similar trend as its CV result, but DPV proved itself more sensitive (Figure 4b). The peak current decreased at every step, from the blank measurement to BSA functionalisation. The peak current of the blank electrode was 345 µA, and then the peak current was measured as 329 µA after Pyr-NHS treatment. The peak current diminished to 303 µA when the antibodies were immobilised. After the final step with BSA, the peak current became 255 µA. These consistent reductions in peak current levels support that the stepwise functionalisation was successfully achieved, as each step caused steric and electrostatic barriers.

#### 3.2.2. Electrochemical Characterisation of Aβ40 Biosensor

The CV result of the biosensor functionalised to detect Aβ40 peptides was parallel to the results of the Aβ42 biosensor (Figure 4c). The highest anodic peak current belonged to the blank measurement with 159 µA, followed by the linker (153 µA), the antibody (147 µA), and the blocking agent (141 µA). The cathodic peak current levels also reduced after every step. The peak cathodic current of the blank electrode was −211 µA. After linker treatment, the cathodic peak was −209 µA. The peak current decreased to −200 µA after antibody immobilisation. In the final step with BSA, the cathodic peak current became −196 µA. These numbers indicate that CV was not sensitive enough to differentiate the electroactivity changes on the electrodes.

The DPV result, however, gave a sensitive response to the electroactivity changes on the electrode for the Aβ40 Biosensor (Figure 4d). The peak current of the blank electrode was 358 µA. With Pyr-NHS, the peak current became 309 µA. Antibody immobilisation caused approximately a 4 µA decrease in the peak current, reaching 305 µA. Finally, the peak current reduced to 269 µA after adding BSA. These responses proved that the functionalisation of the 3D graphene electrode for Aβ40 peptide detection was successful.

### 3.3. Analytical Performance of the Biosensor

**Figure 5:**
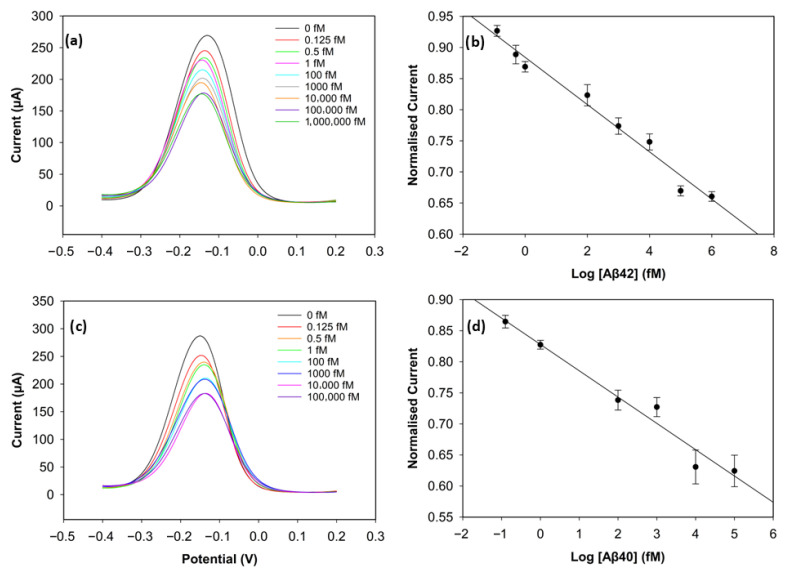
Analytical performance of the biosensor. (**a**) DPV curves of Aβ42 from 0 fM to 1,000,000 fM. (**b**) Calibration curve of Aβ42 with normalised current versus concentration levels in log scale (*n* = 3). (**c**) DPV curves of Aβ40 from 0 fM to 100,000 fM. (**d**) Calibration curve of Aβ40 with normalised current versus concentration levels in log scale (*n* = 3).

To evaluate the analytical performance of the biosensors, DPV was used. Figure 5a,c depict the current values of the wide range of Aβ42 (0 fM, 0.125 fM, 0.5 fM 1 fM, 100 fM, 1000 fM, 10,000 fM, 100,000 fM, 1,000,000 fM) and Aβ40 (0 fM, 0.125 fM, 1 fM, 100 fM, 1000 fM, 10,000 fM, 100,000 fM) concentrations. The calibration plots of the concentrations on a log scale and the normalised current (I_c_/I_blank_) are shown in Figure 5b,d. The normalised current values of both biomarkers decreased linearly as the log concentration increased. It can be seen in Figure 5a,c that saturation was reached after 100 pM for Aβ42 and 10 pM for Aβ40, since there were not enough antibodies left on the electrode surface to catch the biomarkers [11].

The calibration plot of the normalised current versus the logarithm of Aβ42 concentration exhibited a strong linear correlation, described by the regression equation:

I_Aβ42_ = −0.0385log_10_(C) + 0.8817 (1)

with a correlation coefficient of R^2^ = 0.96. The regression equation for Aβ40 was obtained in the same way and was described as follows:

I_Aβ40_ = −0.0407log_10_(C) + 0.7927 (2)

with a correlation coefficient of R^2^ = 0.97. The limit of detection (LOD), determined as 3 × Standard Deviation of Blank/Slope, was 0.252 fM (252 aM) for Aβ42 and 0.395 fM (395 aM) for Aβ40. The reproducibility of the biosensor was assessed by calculating the relative standard deviation of three different electrodes at each concentration under the same conditions. The biosensor demonstrated very good reproducibility for both biomarkers, since the relative standard deviation stayed below 5% during the experiments, indicating high consistency and reliability. This excellent sensing performance of the biosensor may result from the large surface area of the 3D graphene foam, which increases the number of antibodies on the electrode surface [17], and the well-designed binding strategy. The non-covalent π-π stacking of pyrene on the graphene foam surface considerably preserves the material’s inherently high conductivity, while the bond between the NHS ester and the antibodies ensures stability in biorecognition [24].

#### 3.3.1. Optimisation of Antibody Concentration

**Figure 6:**
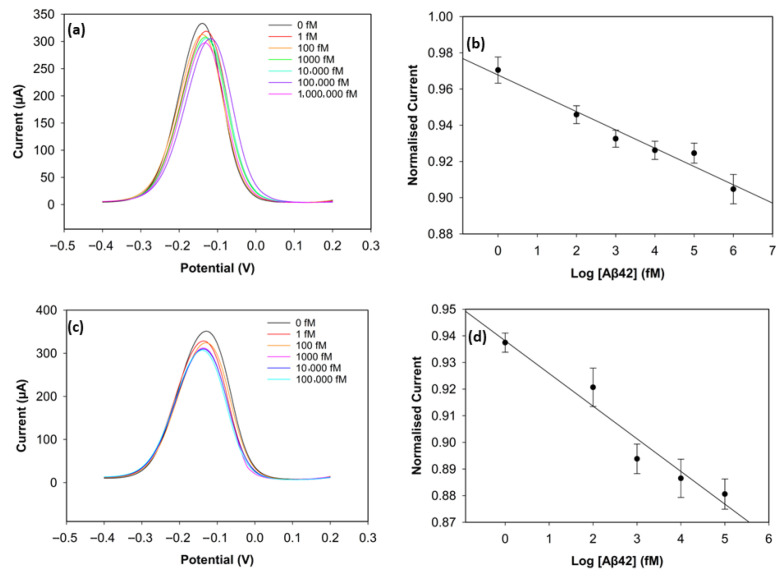
DPV result of Aβ42 with 20 µg/mL of antibody concentration (**a**) and its calibration curve (**b**). DPV result of Aβ42 with 40 µg/mL of antibody concentration (**c**) and its calibration curve (**d**) (*n* = 3).

**Figure 7:**
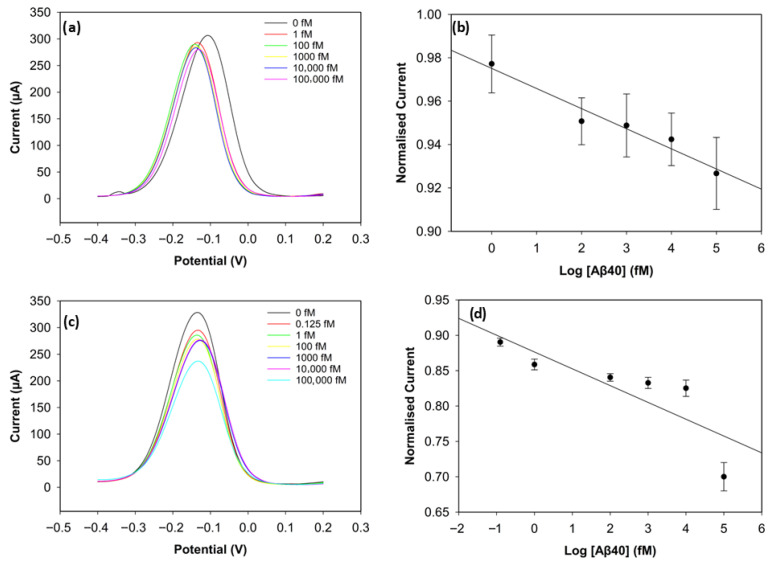
DPV result of Aβ40 with 20 µg/mL of antibody concentration (**a**) and its calibration curve (**b**). DPV result of Aβ40 with 40 µg/mL of antibody concentration (**c**) and its calibration curve (**d**) (*n* = 3).

To improve the sensing performance of the biosensor, the concentration level of the antibody was optimised (Figure 6 and Figure 7). Antibody concentrations of 20 µg/mL, 30 µg/mL, and 40 µg/mL were used. Although the biosensors immobilised with 20 µg/mL antibodies demonstrated clear linearity (R^2^ = 0.96 for Aβ42 and R^2^ = 0.95 for Aβ40), their sensitivity and reproducibility were relatively worse, likely due to insufficient antibody density. The biosensors with 40 µg/mL antibodies showed good reproducibility for Aβ40, but their linearity declined for both antigens (R^2^ = 0.94 for Aβ42 and R^2^ = 0.69 for Aβ40). The sensitivity of the biosensor was unsatisfactory, and this can result from unwanted antibody aggregation on the working electrode, apart from covalent attachment via Pyr-NHS linkers [25]. On the other hand, the biosensors immobilised with 30 µg/mL antibodies, depicted in Figure 5, showed better sensitivity, linearity, and reproducibility.

#### 3.3.2. Additional Immunosensor Optimisation Experiments

To determine the optimum BSA concentration, 0.25% and 1% of BSA were prepared in PBS, and their effects on the linearity and reproducibility of the biosensor’s biorecognition performance were examined. A wide range of antigen (Aβ42) concentrations, including 1 fM, 100 fM, 10,000 fM, and 1,000,000 fM, was prepared, and Differential Pulse Voltammetry (DPV) was conducted to obtain the calibration curves. The calibration curve (Figure S3a) obtained with 0.25% BSA showed that the reproducibility became worse as the antibody concentration increased, although linearity remained high (r^2^ = 0.97). This situation can be attributed to insufficient blocking and heterogeneous BSA coverage. With 1% BSA (Figure S3b), the biosensor showed better reproducibility but worse linearity (r^2^ = 0.86). Biosensors functionalised with 0.5% BSA exhibited the best balance between reproducibility and linearity (Figure 5).

For optimising the antigen incubation time, the analytical performance of the biosensor was compared using 30-min and 120-min antigen incubation periods. Different antigen (Aβ42) concentrations were prepared (1 fM, 100 fM, 10,000 fM, and 1,000,000 fM), and the peak current values were obtained from DPV. The biosensors incubated for 30 min (Figure S4a) with antigen exhibited a high linearity (r^2^ = 0.95). However, the reproducibility was observed to be low, and this can be caused by insufficient time for stable binding. With a 120-min antigen incubation (Figure S4b), the biosensor demonstrated higher reproducibility and good linearity (r^2^ = 0.93). Comparing the analytical performance of the biosensor with different antigen incubation times, biosensors incubated for 60 min for antibody capturing showed a superior performance (Figure 5).

The optimisation outcomes for antigen incubation time and BSA concentration for AB42 were applied to Aβ40 because of their similarities in structure and antibody recognition mechanisms. This approach is consistent with optimisation strategies addressed in recent biosensor studies for detecting Aβ42 and Aβ40, where one biomarker was used to specify the conditions for other biomarkers with similar surface interaction characteristics [11,26].

### 3.4. Specificity and Selectivity Experiments

**Figure 8:**
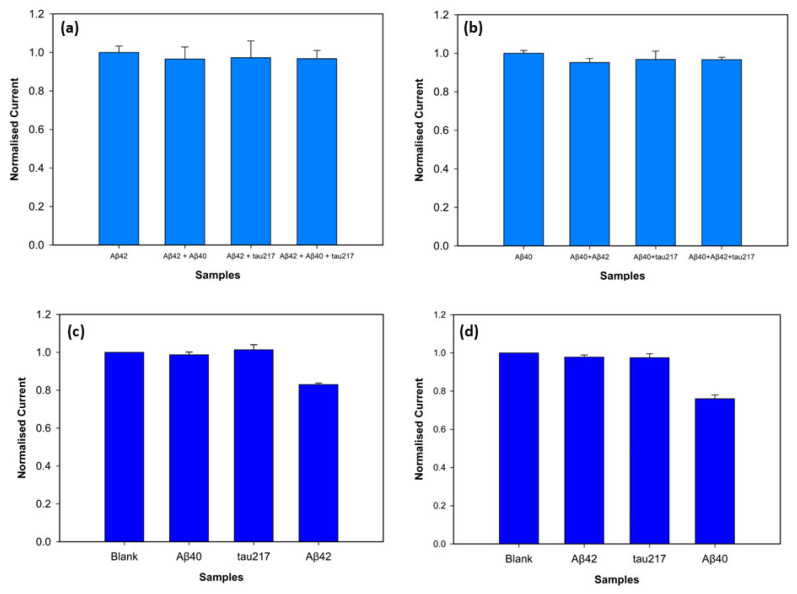
Specificity of the biosensors for Aβ42 (**a**) and Aβ40 (**b**) detection at 100 pM concentrations with the presence of the non-specific antigens at 1 nM concentrations (*n* = 3, *p* < 0.05). Selectivity of the biosensor for detection of 100 fM Aβ42 (**c**) and Aβ40 (**d**) in comparison with the interfering antigens at 1 pM.

To assess specificity of the biosensor, the electrochemical response towards the target antigens was evaluated in the presence of interfering antigens. The target antigens and non-specific antigens were mixed with a vortex mixer. P-tau217 was picked as a non-specific antigen for specificity and selectivity experiments since it is a particularly promising indicator of AD and is found in blood with amyloid beta biomarkers, consistently demonstrating greater efficiency compared to other p-tau isoforms [27]. Peak current values were obtained with DPV, and the normalised peak current values are depicted in Figure 8. Three electrodes were used for each measurement, with 100 pM target antigen and 1 nM non-specific antigens. Figure 8a shows the biosensors incubated with Aβ42 target antigen and the mixtures Aβ42-Aβ40, Aβ42-tau217, and Aβ42-Aβ40-tau217 at a 1:10 concentration ratio. The results showed that the normalised peak current values for Aβ42 and the mixtures Aβ42- Aβ40, Aβ42-tau217, and Aβ42-Aβ40-tau217 showed 96.01%, 97.11%, and 96.78% similarity, respectively. For statistical analysis, a *t*-test confirmed no significant differences between the Aβ42 response, and the responses obtained in the presence of Aβ40 (*p* = 0.387), tau217 (*p* = 0.689), and Aβ40-tau217 (*p* = 0.575). To assess the specificity of the biosensor with Aβ40, the non-specific species, Aβ42 and tau217, were used at a 1:10 concentration ratio. Figure 8b shows the normalised peak current values of Aβ40 as the target antigen and the mixtures Aβ40-Aβ42, Aβ40-tau217, and Aβ40-Aβ42-tau217. The peak currents showed that Aβ40-Aβ42, Aβ40-tau217, and Aβ40-Aβ42-tau217 mixtures showed 95.2%, 96.8%, and 96.72% similarity to the Aβ40 response, respectively. The *t*-test further showing no significant difference between them (*p* = 0.133 for the mixture of Aβ40-Aβ42, *p* = 0.558 for the mixture of Aβ40-tau217, and *p* = 0.147 for the mixture of Aβ40-Aβ42-tau217).

The assay fabricated on the 3D graphene electrode demonstrated excellent selectivity against non-specific biomarkers. The target antigens were tested at a low concentration of 100 fM, whereas the non-specific antigens were prepared at a 10-fold higher concentration of 1 pM to test the rigorousness of the biosensor. Figure 8c shows the results when Aβ42 is the target antigen. The normalised peak current value of Aβ42 was statistically distinct from the background blank value (*p* = 0.0013), while the non-specific antigens Aβ40 (*p* = 0.67) and tau217 (*p* = 0.46) showed no statistical difference from the blank. Figure 8d shows the selectivity for Aβ40 antigen, confirming the statistical difference between the blank and Aβ40 peak current values (*p* = 0.004). The non-specific target antigens, Aβ42 (*p* = 0.19) and tau217 (*p* = 0.35), demonstrated no statistical difference from the blank measurement. These results support that the biosensor successfully discriminates between non-specific antigens and target antigens (Aβ42 and Aβ40) despite high concentrations of non-target antigens.

### 3.5. Stability Experiments

**Figure 9:**
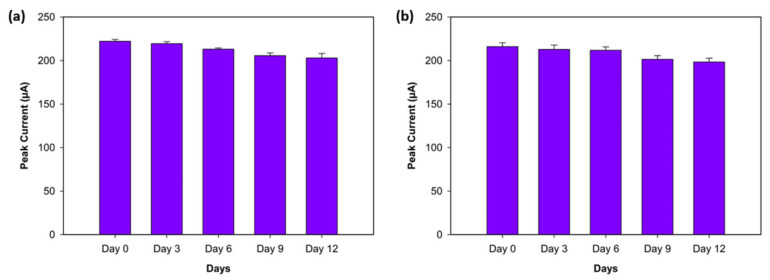
Stability experiment for Aβ42 (**a**) and Aβ40 (**b**) on Day 0 (the first day), Day 3, Day 6, Day 9, and Day 12 (*n* = 3).

The stability of the biosensor was assessed by monitoring the peak current values of three replicates, obtained with DPV, for both antigens (Aβ40 and Aβ42) over a 12-day period while keeping the biosensors under dry conditions at +4 °C. The peak current responses were recorded on Day 0 (the first day), Day 3, Day 6, Day 9, and Day 12. The Aβ42 biosensor showed high stability, exhibiting decreases of 1.25%, 4.04%, 7.4%, and 8.66% on Days 3, 6, 9, and 12, respectively (Figure 9a). Similarly, the Aβ40 biosensor demonstrated minor declines of 1.5%, 1.96%, 6.76%, and 8.2% from Day 3 to Day 9 (Figure 9b). These results support that both biosensors show excellent stability for Aβ42 and Aβ40 over a 12-day period.

### 3.6. Spiked-Diluted Plasma Experiments

**Figure 10:**
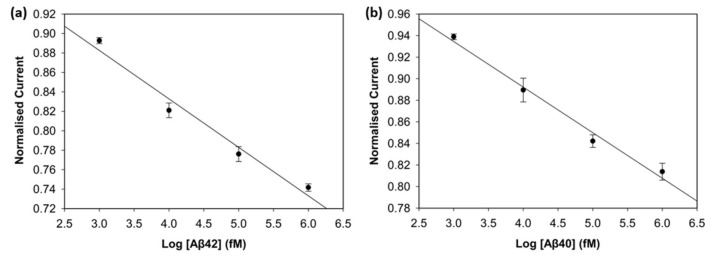
Calibration curve of Aβ42 (**a**) and Aβ40 (**b**) in spiked human plasma (*n* = 3).

To evaluate the analytical performance of the biosensor for Aβ42 and Aβ40 in human plasma, DPV was used. Desired concentration levels were obtained by spiking human plasma with 1 pM, 10 pM, 100 pM, and 1 nM of Aβ42 and Aβ40. Spiked-diluted plasma with no target antigen was used as a background blank. Figure 10 shows the normalised current values versus the log scale of the biomarker concentration. The biosensor demonstrated clear linear correlations for Aβ42 (R^2^ = 0.97) and Aβ40 (R^2^ = 0.98), with impressive reproducibility. Sensitivity was calculated as 14.4 µA/log(fM) for Aβ42 and 14.2 µA/log(fM) for Aβ40. The LOD values were calculated as 1.37 fM for Aβ42 and 1.46 fM for Aβ40. The DPV voltammograms are shown in Figure S2. These results support that the biosensor maintains high sensitivity in a complex biological matrix. The biosensors showed excellent analytical performance in 1:100 spiked-diluted plasma due to reduced matrix effects and preserved analyte integrity. Plasma dilution decreased plasma protein concentrations that could cause non-specific bindings. EDTA, the most commonly used anticoagulant in amyloid beta and tau protein studies, provides stable, non-clotting samples and preserves the target biomarkers in the target analyte [28,29].

## 4. Discussion

The present study demonstrates that the three-dimensional (3D) graphene foam electrode with Pyr-NHS functionalisation provides a robust electrochemical biosensing tool to detect Alzheimer’s Disease (AD) blood biomarkers. The successful covalent coupling between the antibodies and Pyr-NHS linkers, and the stabilisation of the biosensor with BSA as a blocking agent, resulted in a well-defined electrochemical interface, which was confirmed by FTIR and DPV results. Optimising the antibody concentration enhanced the sensing performance of the biosensor. The density of the antibody on the electrode surface affected the sensitivity, linearity, and LOD considerably, which proves that optimising the antibody concentration plays a key role in detecting AD blood biomarkers.

The selectivity and LOD levels achieved with this biosensor surpass those of other conventional two-dimensional graphene-based electrochemical biosensors reported previously. For instance, electrodes with reduced graphene oxide (rGO) showed limitations in antibody immobilisation efficiency [11]. In contrast, the 3D graphene framework used here enabled higher antibody immobilisation and more uniform orientation, resulting in an improved biorecognition process. These features not only provided better detection performance but also ensured biosensor selectivity in the presence of tau217 as an interference agent.

The proposed biosensor exhibited analytical performance in PBS and spiked-diluted human plasma, which is considerably competitive with recent state-of-the-art biosensors (Table 1). Sethi et al. [11] proposed screen-printed electrodes with rGO to detect Aβ42 and Aβ40 biomarkers, and their biosensor showed impressive LOD and sensitivity. The biosensor reported in this article, however, surpassed the sensing performance of that previous study. The biosensor exhibited high selectivity and specificity in the presence of non-specific antigens. The stability test confirmed that the electrodes stayed stable with less than 5% RSD over six days and 10% over twelve days. This results from the optimised antibody concentration, the advanced electrochemical conductivity of 3D graphene foam, and its improved effective electrode surface area. The analytical performance of the biosensor toward Aβ42 and Aβ40 biomarkers is promising both in terms of sensitivity in PBS/spiked-diluted plasma and applicability within the practical concentration range of real samples.

To compare the analytical performance of the biosensors in PBS and spike-diluted plasma, LODs for Aβ42 and Aβ40 in spike-diluted plasma were slightly lower because of complex matrix components in plasma. However, the dilution step and K_2_EDTA as an anticoagulant significantly minimised plasma interference, resulting in femtomolar detection sensitivity for both Aβ42 and Aβ40. Spiked-diluted plasma was used instead of serum since plasma can preserve the concentration of the AB biomarkers with effective anticoagulants, such as EDTA [28,29]. However, during serum preparation, coagulation can occur, leading to the loss of target antigens in the serum. Nonetheless, both plasma and serum are widely used for AD biomarker detection, and it is suggested that biomarker dynamics should be tested independently for both [35].

DPV offered greater sensitivity than CV for monitoring the sequential functionalisation steps in the biosensor. More distinct, quantifiable peak current variations were observed in DPV, which provides easier differentiation between each step: from the blank electrode to Pyr-NHS functionalisation, to antibody immobilisation, to BSA coverage, and finally to antigen capture. DPV offers minimised capacitive current, resulting in better signal resolution [21].

Despite these promising results, several limitations need to be addressed. The biosensor was mainly evaluated in a controlled buffer system and spiked human plasma (1:100 dilution ratio in PBS), which do not fully replicate human serum. Additionally, the analytical performance needs to be tested at lower antigen concentrations (1 fM–1 pM), and different dilution ratios (1:10, 1:50) should be compared. In the experiments, three replicas were used; to improve statistical robustness, more replicas should be used. Additionally, long-term stability tests remain as a challenge and need further investigation. Although electrochemical measurements (CV and DPV) exhibited well-defined stepwise functionalisation of the biosensor, FTIR remained insensitive to show surface modification changes, which is likely because of the small amount of biological and chemical material accumulated onto the 3D graphene foam. Thus, more sensitive surface-specific characterisation methods, such as XPS and ToF/SIMS, should be used in future work.

In addition to the present findings, monitoring amyloid beta dynamics in human serum using 3D graphene foam electrodes will be an important future direction. Generally, electrochemical biosensors for AD focus on the detection of a single biomarker, whereas clinical studies concentrate on the diagnostic significance of the Aβ42/Aβ40 ratio [36,37,38]. By preparing samples in human serum that reproduce the clinical Aβ42/Aβ40 ratio observed in patients and healthy people, it will be possible to evaluate the clinical relevance of the biosensor more closely.

The present study highlights the significant benefits of 3D graphene foam electrodes combined with rational surface functionalisation and antibody concentration optimisation for achieving sensitive, selective, and adaptable biosensors. The study also shows the promise of 3D nanostructured electrodes as a foundation for the next generation of electrochemical biomarker detection.

## 5. Conclusions

In summary, a 3D graphene foam-based electrochemical biosensor was developed and characterised for AD blood biomarkers (Aβ42 and Aβ40). Through antibody immobilisation via Pyr-NHS and BSA blocking, the biosensor achieved selective and sensitive detection with low limits of detection, with tau217 protein used to validate specificity. The spiked-diluted plasma analysis further supported the validation of the biosensor’s performance. These findings highlight the advantages of 3D graphene foam electrodes—enhanced surface area, high conductivity, and strong biomolecular interaction—the effectiveness of Pyr-NHS for stable functionalisation, and the optimisation of antibody density on the working electrode surface, making the system promising for ultrasensitive AD biomarker detection. Future work should focus on detecting Aβ42 and Aβ40 peptides in human serum with the clinical detection ratio of Aβ42/Aβ40.

## Figures and Tables

**Table 1:** Comparison of the present study with recent studies.

Study (Electrode/Material)	Biomarkers (Detected)	Linear Range	Limit of Detection	Sample Matrix	Reference
3D graphene foam electrode modified with Pyr-NHS	Aβ42/Aβ40	0.125 fM–1 nM for Aβ42/ 0.125 fM–100 pM for Aβ40	252 aM for Aβ42 in PBS, 1.37 fM in spiked-diluted plasma/ 395 aM for Aβ40 in PBS, 1.46 fM in spiked-diluted plasma	PBS and spiked-diluted plasma	This work
SPGE modified with p-DAN (polymerised 1,5-diaminonaphthalene)	Aβ42	1 pg/mL to 1000 pg/mL	~0.31 pM	Spiked-diluted plasma	[30]
Screen-printed rGO modified with NH_2_	Aβ42/Aβ40	5 fM–100 pM for Aβ42 5 fM–50 pM for Aβ40	8.65 fM for Aβ42 in PBS, 9.51 fM for Aβ40 in PBS	PBS and spiked-diluted plasma	[11]
CNT-Au nanostar PEG device	Aβ42/ApoE4	15.63 pg/mL–1000 pg/mL for Aβ42	~0.3 pM	Plasma	[31]
Microporous gold nanostructure with Aβ-binding peptide on Au	Aβ42	3 pg/mL to 7000 pg/mL	~44 fM	Spiked serum	[32]
Vertical Graphene@nanoAu printed electrode array (VG@nanoAu)	Aβ42, Aβ40, T-tau, P-tau181	0.1 pg/mL–10 pg/mL	~20 fM Aβ42, ~17 fM for Aβ40	Serum	[33]
Fern leaves-like gold nanostructure immobilised with RNA	Aβ42	2 pg/mL–1280 pg/mL	~89 fM	Artificial CSF and serum	[34]
Gold nanoparticle/nickel ferrite/graphene oxide–chitosan nanocomposite-modified glassy carbon electrode	Aβ42	1 pg/mL–1 ng/mL	0.66 pM	PBS and CSF	[12]

## Data Availability

The original contributions were included in the article. Further inquiries can be directed to the corresponding author.

## Supplementary Materials

The following supporting information can be downloaded at: https://www.mdpi.com/article/10.3390/bios15120806/s1. Figure S1: Picture of the Gii-Sens Integrated Graphene electrode with its three-electrode platform (Working Electrode, Reference Electrode, and Counter Electrode). Figure S2: Voltammogram of the biosensor with Aβ42 (a) and Aβ40 (b) obtained with the spiked-diluted plasma experiments (1:100 in PBS). Figure S3: Calibration curve of the Aβ42 biosensors functionalised with 0.25% of BSA (a) and 1% of BSA (b) (*n* = 3). Figure S4: Calibration curve of the Aβ42 biosensors with 30-min antigen incubation (a) and 120-min antigen incubation (b) (*n* = 3).
