# Supplementary material for: Electrochemical Detection of Aβ42 and Aβ40 at Attomolar Scale via Optimised Antibody Loading on Pyr-NHS-Functionalised 3D Graphene Foam Electrodes
Source: Biosensors (Basel). 2025 Dec 10;15(12):806. doi: 10.3390/bios15120806 (PMC12731046; doi:10.3390/bios15120806)
Supplement: Supplementary file 1 [file biosensors-15-00806-s001.zip › biosensors-3980797-supplementary.pdf]

Supporting Information

Electrochemical Detection of Aβ42 and Aβ40 at Attomolar Scale via Optimised Antibody Loading on Pyr-NHS-Functionalised 3D Graphene Foam Electrodes

Muhsin Dogan [1,2,3*], Sophia Nazir [1,3], David Jenkins [1,3], Yinghui Wei [3] and Genhua Pan [1,3]

1 Nanomaterials and Devices Laboratory (NMD), School of Engineering, Computing and Mathematics, University of Plymouth, Devon, PL4 8AA, UK

2 Biomedical Engineering, Engineering and Architecture Faculty, Izmir Bakircay University, Izmir 35665, Turkey

3 School of Engineering, Computing and Mathematics, University of Plymouth, Devon, PL4 8AA, 7 UK

* Correspondence: muhsin.dogan@plymouth.ac.uk. doganmuhsin.01@gmail.com

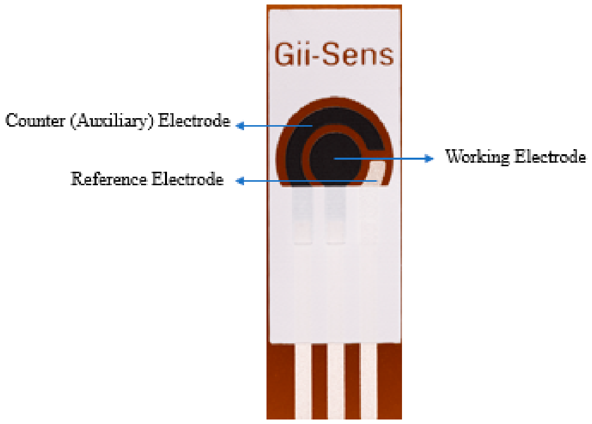

Figure S1: The picture of the GiiSens Integrated Graphene electrode with its three-electrode platform (Working Electrode, Reference Electrode and Counter Electrode.

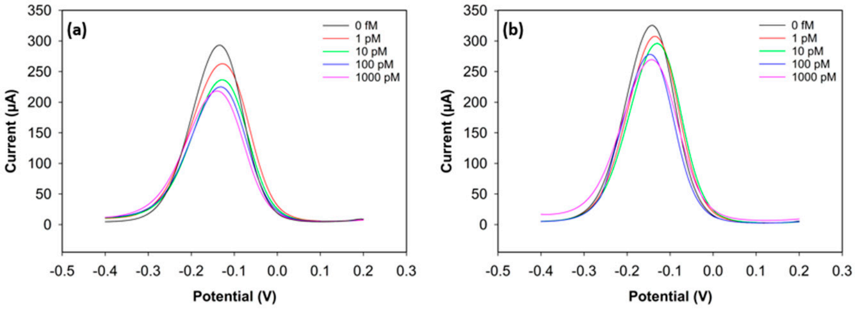

Figure S2: The voltammogram of the biosensor with Aβ42 (a) and Aβ40 (b) obtained with the spiked diluted plasma experiments (1:100 in PBS).

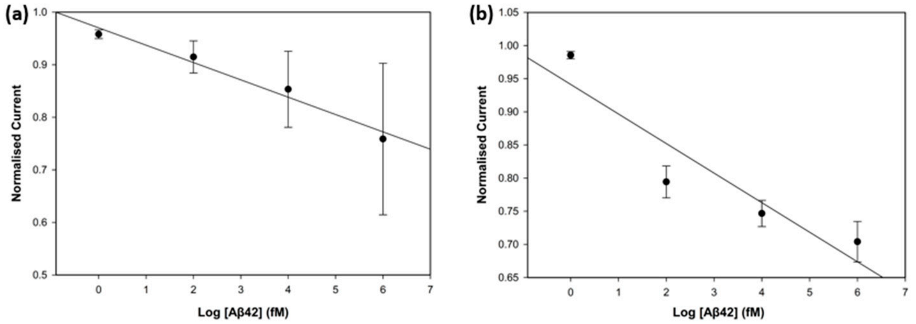

Figure S3: The calibration curve of the Aβ42 biosensors functionalised with 0.25% of BSA (a) and 1% of BSA (b) (n=3).

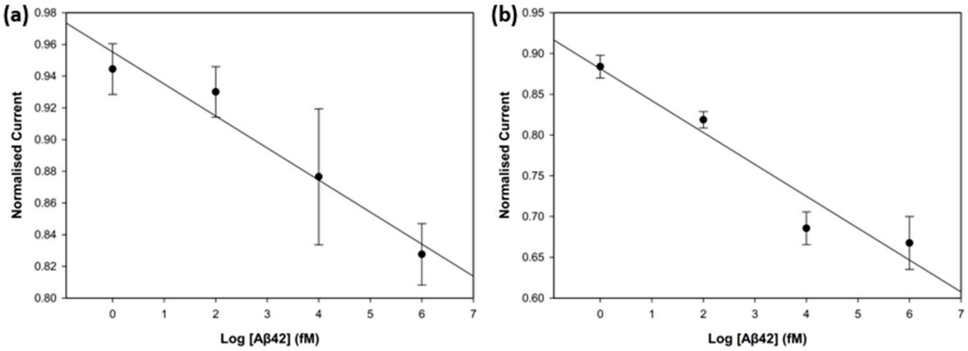

Figure S4: The calibration curve of the Aβ42 biosensors with 30-minute antigen incubation (a) and 120-minute antigen incubation (b) (n=3).
